# Spike-wave discharges are uncoupled with cognitive outcome in aged Long-Evans rats

**DOI:** 10.1007/s00213-025-06976-8

**Published:** 2025-12-10

**Authors:** Craig Myrum, Zach Uttke, Diba Ramezan, Margaret E Stelzner, Jeffrey M. Long, Peter R. Rapp

**Affiliations:** 1 Laboratory of Behavioral Neuroscience, Neurocognitive Aging Section, National Institutes of Health (NIH), National Institute on Aging, Baltimore, MD USA; 2 Department of Biology, Loyola University Maryland, 4501 N. Charles St, Baltimore, MD 21210 USA

**Keywords:** SWD, Cognitive aging, Cognitive outcome, Long-Evans rats, Hippocampus, Antiepileptic drugs, Levetiracetam, Ethosuximide

## Abstract

**Rational:**

Rodent EEGs display 7–12 Hz oscillations called spike-wave discharges (SWDs) that are more abundant with age, and some data suggest that they might interfere with hippocampus-dependent memory processing.

**Objective:**

To test whether SWD activity contributes to the memory decline reported in aged rats.

**Methods:**

Spatial learning was assessed in young and aged Long-Evans (LE) rats using a water maze protocol where aged rats display substantial interindividual variability in hippocampus-dependent memory. We then collected 24 h EEG data to quantify SWD incidence and duration among young and aged animals. Lastly, we took a pharmacological approach to test the efficacy of two antiepileptic drugs—levetiracetam and ethosuximide—at reducing SWD activity.

**Results:**

We found that SWDs were significantly more frequent in aged rats than young, but entirely unrelated to between-subject differences in cognitive outcome. Age-dependent increases in SWD frequency were also insensitive to levetiracetam at doses known to reverse memory impairment in aged rats, but potently inhibited by the other antiepileptic drug, ethosuximide, that functions through a different mechanism of action.

**Conclusion:**

Together these findings provide evidence that the disrupted excitatory/inhibitory balance associated with individual differences in cognitive aging is unrelated to the SWD increase seen in aged rats.

## Introduction

Rodent models have been a valuable source of insight into the underlying mechanisms responsible for age-related cognitive impairment. Studies in aged rats, for example, demonstrate that elevated neural activity within specific components of hippocampal circuitry is coupled with deficits in spatial learning and memory (Wilson et al. 2003; Wilson 2005). Prompted by this background, related findings in monkeys (Thomé et al. 2016) and humans (Yassa et al. 2010) have confirmed that hippocampal hyperactivity is a prominent signature of poor cognitive outcome in aging. Pharmacological treatment targeting this excess activity provides a means of probing the direction of effect, testing whether it contributes to cognitive deficits in aging, or alternatively, represents a failed compensatory mechanism. For example, low doses of the antiepileptic drug (AED) levetiracetam (LEV) reportedly reverse cognitive deficits in human amyloid precursor protein (hAPPJ20) AD mice (Sanchez et al. 2012), as well as in a model of naturally occurring cognitive aging in LE rats (Koh et al. 2010), i.e., two conditions featuring neuronal hyperactivity.

Electroencephalogram (EEG) recordings in rats and mice often reveal prominent 7–12 Hz oscillations known as spike-wave discharges (SWDs) that are usually brief (1–2 s), terminate abruptly, and frequently occur together with vibrissa twitching during quiet wake. Some authors have suggested that these paroxysmal episodes resemble absence seizures in humans—both in terms of duration (on the scale of seconds) and associated immobility (Robinson and Gilmore 1980; Vergnes et al. 1982), though the precise temporal characteristics differ. Selective inbreeding for SWDs in Wistar rats yielded a widely used model of absence epilepsy, namely the Genetic Absence Epileptic Rat from Strasbourg (GAERS), and the observation that Wistar Albino Glaxo rats from Rijswijk (WAG/Rij) consistently display SWD activity has led to their wide-spread use as a model of absence epilepsy (Depaulis and van Luijtelaar 2006). Compared with age-matched controls, WAG/Rij and GAERS rats display impaired episodic memory (Karson et al. 2012; Marques-Carneiro et al. 2016; Leo et al. 2019), potentially related to the disruptive effect of SWDs on hippocampal activity (Nersesyan et al. 2004; Pearce et al. 2014). SWDs are also commonly observed in various strains of laboratory rats, including Long-Evans (LE) (Taylor et al. 2019), where the number and duration of SWDs increases markedly with age (Coenen and Van Luijtelaar 1987; Rodgers et al. 2015). What has not been determined is whether SWDs contribute to the well-documented hippocampal hyperactivity and associated spatial memory impairment observed in this model of cognitive aging.

Here we directly examined if the number and duration of SWD episodes is coupled with cognitive aging in Long-Evans rats, leveraging a well-characterized model (Gallagher et al. 1993) featuring substantial individual differences and increased hippocampal activity associated with age-related spatial memory deficits. In subsequent pharmacological experiments, we used a within-subject design to compare the efficacy of two antiepileptic drugs in reducing SWDs; LEV at doses known to improve memory performance in aged rats when administered chronically (Koh et al. 2010), and another anticonvulsant, ethosuximide (ETX), with known clinical efficacy in absence epilepsy (Glauser et al. 2010), but that functions by a different mechanism of action (Coulter et al. 1989; Lynch et al. 2004). To the degree that SWDs contribute to the hyperactivity linked with age-related memory impairment in the LE rat model, we reasoned that both low-dose LEV and ETX should both be effective in blunting SWDs in aged rats with memory impairment.

## Methods

### Subjects and experimental design

Outbred male Long-Evans rats (Charles River Laboratories, Wilmington, MA, USA; Raleigh, NC colony) were housed individually and maintained on a 12:12 h light: dark cycle with food and water *ad libitum.* Subjects comprised young controls (6–8 months, *n* = 5) and a larger sample of aged rats (24–26 months, *n* = 31) that were behaviorally tested in a standardized water maze task (see below), providing background cognitive characterization for two experiments. In the first study, EEGs were recorded from both young (*n* = 5) and aged animals (*n* = 12), and the number and duration of SWDs over 24 h was quantified (see below). A second, follow-up experiment examined the effects of AEDs on SWD frequency and duration in the remaining aged rats (*n* = 19). Since the pre-drug, baseline assessment of SWDs in the AED cohort was identical across experiments, the EEG data from all aged rats (i.e., total *n* = 31) was used to test for potential age effects in comparison with results from the young animals. This design allowed us to test the efficacy of multiple AEDs in blunting SWDs, including doses of LEV that reportedly improve spatial memory impairment in the aged rat model examined here (Koh et al. 2010), and ETX, which does not display this ability (Nygaard et al. 2015). On the view that SWDs contribute to these impairments, the prediction was that AEDs would exhibit corresponding effects on both memory and SWDs in aging. All procedures were approved by the Animal Care and Use Committee of the Intramural Research Program of the National Institute on Aging, in accordance with the National Research Council Guide for the Care and Use of Laboratory Animals.

### Behavioral testing

Spatial learning and memory were assessed using a hippocampus-dependent procedure in the Morris water maze (MWM), where aged LE rats reliably display substantially increased interindividual variability in performance on this task after 20 months of age (Gallagher et al. 1993). The task was carried out identically to many previous reports using this model (e.g. Fletcher et al. 2014; Myrum et al. 2020, 2019; Wołoszynowska-Fraser et al. 2021). Specifically, animals were trained for eight days (three trials per day) to locate a hidden escape platform that remained in the same location throughout the task. Every sixth trial was a probe in which the escape platform was unavailable for the first 30 s of the trial, and accuracy navigating to the escape location was measured via video tracking system (ANY-maze; Stoelting, Wood Dale, IL, USA). A learning index (LI), calculated based on proximity to the platform location during probe trials, was used to distinguish impaired (AI, learning index > 240) from unimpaired (AU, learning index < 240) aged rats (Gallagher et al. 1993). By this measure, low scores reflect better retention and more accurate searching focused on the platform location. Cue training (visible escape platform; six 30 s trials) was conducted on the last day of training to exclude rats with sensorimotor deficits. Following MWM testing, rats remained in their home cage for at least one week prior to subsequent EEG implantation.

### Surgery

A week or more after background behavioral characterization in the water maze, rats were anesthetized using isoflurane in an induction chamber and maintained under 2% through a nosecone while secured in a stereotactic frame (David Kopf Instruments). Ophthalmic ointment was applied to the eyes. The scalp was incised and retracted along the longitudinal midline, and five small skull holes were drilled at the following coordinates relative to bregma: 1) AP: 2 mm; ML 0 mm (for anchoring); 2) AP: −2 mm; ML: 3 mm (for EEG); 3) AP: −5.6 mm; ML: 3 mm (for EEG); and 4–5) AP: 2 mm; ±3 mm (ground and reference). Tight-fitting stainless steel slotted flat head machine screws (0–80 × 1/8”) were lowered into the holes just enough to touch the dura without penetrating the brain. Silver wire soldered to the screw heads connected to an electronic interface board (EIB), held in place with gold-plated pins. Four additional connections were made with the EIB; two coated stainless-steel wires placed in the neck muscle to monitor electromyogram (EMG) activity, and two others immediately behind the eyes for electrooculogram (EOG) activity. These additional recordings were used for a separate study focusing on sleep. Implants were fixed in place with dental acrylic. Antibiotic cream was applied, and the incision was stapled shut. Ibuprofen was administered in the drinking water as a postoperative analgesic, and animals recovered a minimum of seven days prior to further testing.

### Recordings and SWD quantification

Following the > 1 week postoperative recovery period, animals were habituated to a quiet recording chamber for a minimum of three days. EEG, EOG, EMG, and accelerometer activity were recorded for 24 h using Oneiros telemetric/data-logger system (Viewpoint Behavior Technology, Civrieux, France) at a sampling rate of 256 Hz. EEG files were examined manually in 1 s increments to quantify SWD duration and number of SWD occurrences, as similarly done previously (Talnov et al. 2012; Pearce et al. 2014; Born et al. 2014; Nygaard et al. 2015; Frolov et al. 2019). We defined SWDs as events that were at least 1 s in duration, were characterized by sharp, negative spikes followed by positive waves, were at least twice the amplitude of the background EEG signal immediately before and after SWD activity, and were accompanied by muscle hypotonia (Fig. 2a).

### Pharmacology

The incidence and duration of SWDs were measured in aged rats with memory impairment administered a series of either LEV or ETX doses in ascending/descending order, with approximately half receiving ETX doses first and others receiving LEV. The objective was to test each dose twice, though not every animal received all doses due to either mortality or insufficient EEG quality. Both LEV (Tocris) and ETX (Sigma) were diluted in a vehicle solution of 0.9% sterile saline and injected intraperitoneally. LEV doses were 1.25, 10, and 50 mg/kg, while ETX doses were 25, 100, and 200 mg/kg. All rats also received at least one injection of 0.9% sterile saline control. Recordings commenced shortly after injection, at approximately 9AM. We allowed for a minimum 24 h washout of LEV and 48 h for ETX, based on their respective half-lives in rats (10–16 h for ETX; 2–3 h for LEV; Löscher 2007). Following each injection, EEGs were recorded for 3 h and quantified as described above.

### Statistical analyses

Statistical analyses were performed using GraphPad Prism Version 10.2.3. Analyses included one-way ANOVAs with Tukey’s test for multiple comparisons in pair-wise contrasts of SWD activity in Y, AU, and AI rats (Fig. 2), unpaired t-tests to examine SWD activity between Y and aged (AU + AI) rats (Fig. 2), and a post-hoc power analysis using G*Power (Faul et al. 2007). In addition, potential relationships between learning index scores and SWDs were assessed by two-tailed Pearson r correlation coefficients. Repeated measures ANOVAs were used to compare SWD activity across 24 1-hour bins. Finally, linear mixed effects analysis (selected to accommodate missing data for a subset of AED doses; Fig. 3) with Dunnett corrections for multiple comparisons, was used to test for differences in SWD activity following LEV and ETX administration.

## Results

### Morris water maze

**Fig. 1:**
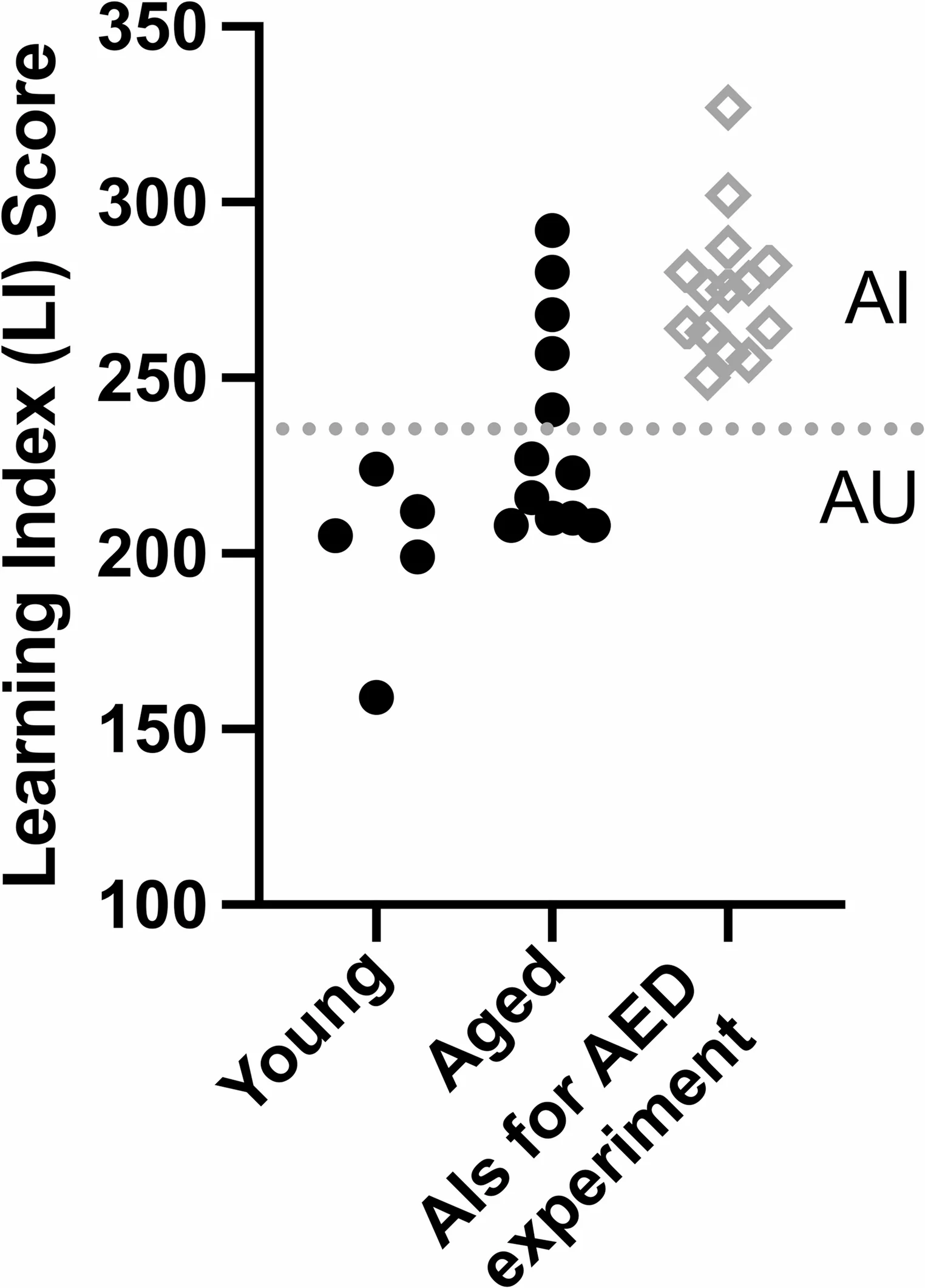
Assessment of young and aged male Long-Evans rats in the Morris water maze (MWM). Distribution of individual subject MWM learning index (LI) scores for young and aged rats as well as aged-impaired (AI) animals used to test the effects of anti-epileptic drugs (AEDs). Aged rats with LI scores above 240 (dotted line) were classified as impaired.

The distribution of individual differences spatial learning in young and aged rats was consistent with many earlier studies (e.g., Fletcher et al. 2014; Myrum et al. 2020, 2019; Wołoszynowska-Fraser et al. 2021). More specifically, while Y animal scores averaged 200 ± 25 (SD; Fig. 1; *n* = 5), performance in aged rats (24–28 months old; *n* = 12) varied considerably around an average of 237 ± 30. Following the convention of previous reports using this model (e.g., Fletcher et al. 2014; Gallagher et al. 1993; Haberman et al. 2017; Myrum et al. 2020, 2019; Wołoszynowska-Fraser et al. 2021), in order to evaluate SWDs in relation to cognitive outcome, aged animals were operationally defined as unimpaired (AU) if their LI scores were on par with Y rats, under 240 (Fig. 1). LI scores for AI rats selected to test the effects of AED administration averaged 275 ± 20 (SD; Fig. 1; *n* = 19).

### SWD quantification in Y, AU, and AI rats

**Fig. 2:**
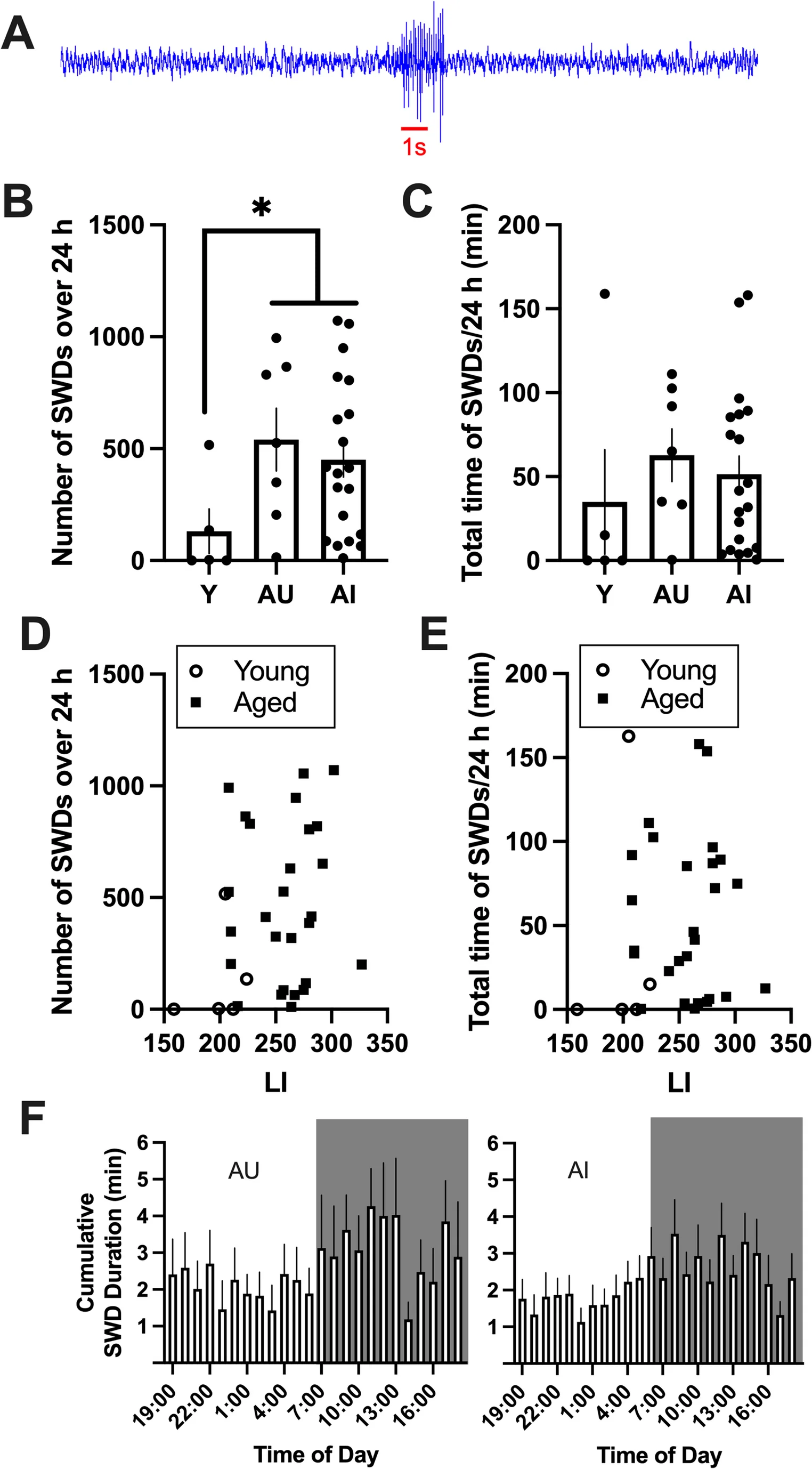
SWD activity in young (Y), aged unimpaired (AU), and aged impaired (AI) Long-Evans rats. (**a**) Representative EEG trace that includes one spike-wave discharge (SWD), indicated above the red line. (**b**) The number of SWD episodes over 24 h was greater among aged animals compared to Y, while Y/AU/AI comparisons were not significantly different. (**c**) The total time spent in the SWD state over 24 h did not significantly differ between Y and aged rats (AU + AI), nor did Y/AU/AI comparisons. (**d**) The number of SWD episodes over 24 h was not correlated with LI scores in neither young nor aged animals. (**e**) The total time spend in the SWD state over 24 h was not correlated with LI scores in neither young nor aged animals. (**f**) SWD activity did not significantly differ over the light/dark cycle in AU rats (left panel) but did among AI rats (right panel). *p < 0.05

We first examined whether SWD activity is coupled to chronological age. These analyses revealed that aged animals (AU + AI) exhibited more SWDs over 24 h than Y animals (Fig. 2b; t_(30)_ = 2.10, *p* = 0.044), while the difference in total time spent in this state between Y and aged rats failed to reach statistical significance (Fig. 2c; t_(30)_ = 0.80, *p* = 0.43). Variability in SWD incidence among the aged rats was pronounced, and to assess whether SWDs are coupled with cognitive outcome in aging, next we compared SWD activity between the Y, AU, and AI subgroups. These comparisons showed that the total number of SWDs over 24 h did not significantly differ between groups overall (Fig. 2b; *F*_(2,29)_ = 2.33; *p* = 0.12), or between AU and AI rats (Fig. 2B; t_(25)_ = 0.57, *p* = 0.57). Numerical differences between the young and aged groups in total time in SWDs were not statistically significant (Fig. 2c; *F*_(2,29)_ = 0.44; *p* = 0.65). A post hoc power analysis was conducted with G*Power, which showed an overall effect size of 0.4, and the power to detect an effect of this size was computed to be 0.47. To reach an overall ANOVA p value of < 0.05, the required sample size was estimated at a total of 102 animals. Correlation analyses confirmed that spatial learning capacity was not coupled to SWD activity; either with respect to the number of SWDs in young (Fig. 2 d; r^2^ = 0.07, *p* = 0.66) or aged animals (Fig. 2 d; r^2^ = 0.002, *p* = 0.85), or the duration of SWD activity in young (Fig. 2e; r^2^ = 0.03, *p* = 0.78) or aged animals (Fig. 2e; r^2^ = 0.001, *p* = 0.87). While time of day had no effect on SWD activity among AU animals (Fig. 2f; F_(4.060, 24.36)_ = 1.232, *p* = 0.3233), SWD activity among AI animals significantly differed depending on time of day (Fig. 2f; F_(5.584, 100.5)_ = 2.289, *p* = 0.0451), where activity was higher during the dark (active) period than their own activity during the light (sleep) period. Nonetheless, amount of SWD activity failed to differ in comparisons between AU and AI subgroups in either the light or dark phases (dark: t_(24)_ = 0.5; *p* = 0.62; light: t_(24)_ = 0.3; *p* = 0.78). Together these data indicate that SWDs are unlikely to account for cognitive deficits observed in aged rats with memory impairment.

### AED effects on SWDs

**Fig. 3:**
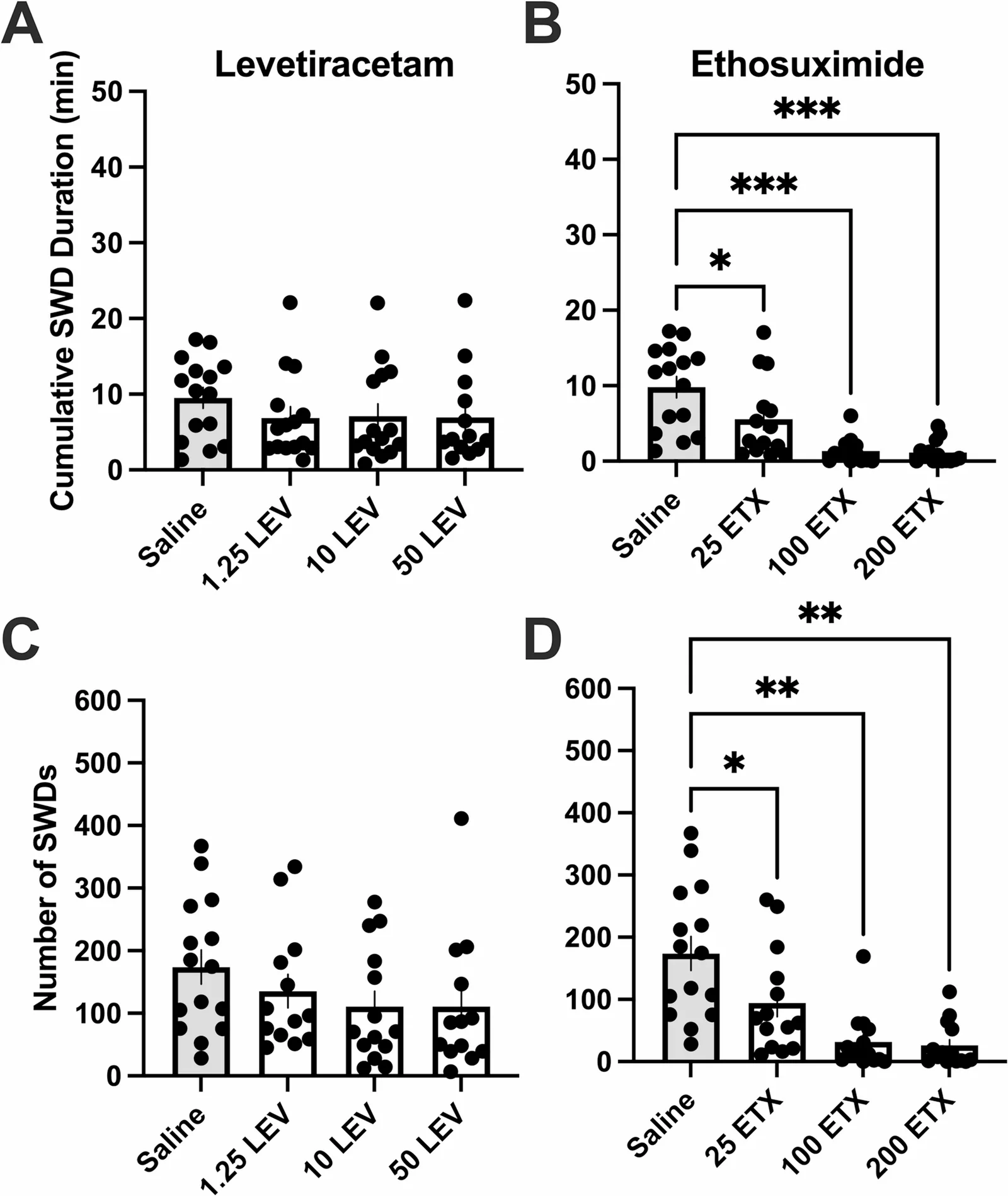
Effects of levetiracetam (LEV) and ethosuximide (ETX) on spike-wave discharge (SWD) activity. (**a**) Acute treatment with LEV did not significantly reduce the total time of SWD activity over 3 h compared to saline controls. (**b**) Acute treatment with ETX significantly reduced the total time of SWD activity over 3 h, across all three doses, compared to saline controls. (**c**) Acute treatment with LEV did not significantly reduce the number of SWD occurrences over 3 h compared to saline controls. (**d**) Acute treatment with ETX significantly reduced the number of SWD occurrences over 3 h, across all three doses, compared to saline controls. *p < 0.05, **p < 0.01, ***p < 0.001

Next we tested the potential contribution of SWDs to cognitive aging using a pharmacological approach, including doses of the AED LEV that improve spatial in impaired aged rats (Koh et al. 2010). While none of the three LEV doses had a significant effect on SWD duration (Fig. 3a; *F*_(2.8,35.5)_ = 1.16; *p* = 0.34) or incidence relative to saline control values (Fig. 3c; *F*_(2.2,26.7)_ = 2.07; *p* = 0.14), ETX significantly reduced both the frequency and amount of time spent in the SWD state (Fig. 3b; *F*_(1.8,21.2)_ = 21.07; *p* < 0.0001) (Fig. 3d; *F*_(1.9,23.04)_ = 16.55; *p* < 0.0001) over the three hours following drug administration. While ETX works by blocking T-type calcium channels (Coulter et al. 1989), LEV’s main mechanism of action is through the interaction with the synaptic vesicle protein 2 A (SV2A), which is a novel mechanism among AEDs (Lynch et al. 2004). Thus, SWD activity was insensitive to LEV at doses known to improve memory in AI rats (Koh et al. 2010), but robustly blunted another AED targeting a different mechanism of action.

## Discussion

As established in numerous earlier studies, outbred LE rats recapitulate the increased variability in cognitive function seen in older humans; i.e. deficits are observed in some aged rats while others at the same chronological age perform on par with young adult animals (Rapp et al. 2020). Consistent with previous studies using the LE model of normal cognitive aging, here we observed substantial interindividual variability in hippocampus-dependent MWM spatial learning in old animals. This feature of the LE rat model of normal cognitive aging has yielded valuable insight into the mechanisms of cognitive aging and resilience. Electrophysiology experiments in LE rats, for example, indicate that higher hippocampal firing rates are associated with spatial learning impairment (Wilson et al. 2003; Wilson 2005), and a large body of subsequent work has established hippocampal hyperactivity as a key indicator of poor cognitive outcome in aging (Leal and Yassa 2015). Here we report that SWD activity, which has been well-documented across multiple rat strains (Taylor et al. 2019), and increases with age in Sprague-Dawley (Rodgers et al. 2015), WAG/Rij (Coenen and Van Luijtelaar 1987), and Fischer 344 rats (Buzsáki et al. 1990), is also elevated in aged LE rats. Prompted by this background, the current study examined the possibility that changes in SWD activity are among the effects of aging on excitatory balance in the aged brain associated with memory impairment.

The first key finding reported here is that both the total amount of time spent in the SWD state, and the total number of SWD episodes, are entirely uncoupled from the integrity of spatial memory in aged LE rats. To the degree that findings from models of absence epilepsy (i.e. GAERS and WAG/Rij) track with the neurobiology of outbred rat strains like LE rats (Taylor et al. 2017), our findings counter the idea that SWDs inherently disrupt neural network dynamics essential for normal memory. In one study, age-related deficits in MWM performance in WAG/Rij rats paralleled increases in SWD activity (Karson et al. 2012). Related research is inconsistent, with one study in the same strain finding that the hippocampus displays marked increases in fMRI signal during SWD activity (Nersesyan et al. 2004), and another reporting no effect of SWDs on neuroimaging signals of hippocampal activity (Mishra et al. 2011). Electrophysiological evidence is more consistent, demonstrating that hippocampal neural activity is acutely affected by SWD activity. In one study, EEG data collected from Sprague-Dawley rats showed that SWD frequency was highly correlated with hippocampal theta frequency (Pearce et al. 2014), while another employing a similar technique found that EEG spectral power markedly increased in the hilus of the hippocampus in both GAERS and WAG/Rij rats during SWDs (Papp et al. 2018). Amid these mixed conclusions, our data suggest that the specific neural network dynamics that are influenced by SWDs may be largely distinct from the changes that contribute to individual differences in hippocampal memory in aging. The timing of active systems memory consolidation lends further support to this interpretation. Specifically, SWDs occur predominantly during quiet wakefulness and N1-N2 sleep, whereas reactivation of recently encoded neuronal memory representations and neocortical transfer occur during N3 slow-wave sleep (Rasch and Born 2013). Indeed, we observed that SWD activity was moderately higher during the dark (active) period, which reached significance among AI animals. Taken together, age-related cognitive decline in these animals is more likely the consequence of the well-documented, region-specific disruptions in excitatory/inhibitory balance and synaptic regulation within aging neural circuits than increased SWD activity.

Complementing and extending the preceding evidence, a second key finding in the current study is that pharmacological treatment known to reverse age-related memory impairment in the model used here failed to significantly affect the observed age-dependent increase in SWDs. Treatment with low (5–10 mg/kg) but not higher doses of LEV (20 mg/kg) has previously been shown to reverse memory impairment LE rats (Koh et al. 2010). Clinical investigation prompted partly by that work tested LEV in patients with mild cognitive impairment (MCI) and found that, similar to results in aged rats, LEV significantly improved cognitive function selectively at low doses (Bakker et al. 2012, 2015). Here we found that 10 mg/kg of LEV, which improves memory in aged impaired LE rats (Koh et al. 2010), failed to significantly suppress either the incidence or duration of SWD activity. While LEV (75 mg/kg) has been shown to decrease the duration and number of SWD events during “mixed” sleep/wake behavioral states, it also conversely increases the number of SWD events during sleep (Jin et al. 2020). The implication of these findings is that the cognitive benefits of LEV in LE rats are likely mediated independently of the mechanisms underlying SWDs. Other studies have reported partial decreases in SWD incidence and duration following LEV treatment in GAERS rats, WAG/Rij rats, and APPswe/PS1dE9 (APP/PS1) mice, roughly by half, particularly at higher doses (Gower et al. 1995; Bouwman and van Rijn 2004; Jin et al. 2020). These divergent outcomes across studies might be attributable to differences in rat strains and associated amounts of genetic variability. By comparison our findings confirm that ETX, which has been shown to potently reduce SWD time and occurrence in APP/PS1 mice (Jin et al. 2020), GAERS (Richards et al. 2003), and LE rats (Kozák et al. 2020), sharply reduces SWD activity across all doses tested. Since LEV and ETX have different mechanisms of action, as described above, their divergent effects imply that some molecular targets may modulate cognition independently of SWD activity.

Alongside research probing the neurophysiological effects of AEDs, studies investigating ETX as a pharmacological treatment to improve memory have reported mixed results. For example, while wild-type adult Wistar rats treated with ETX (125 mg/kg; i.p.) performed significantly better than saline controls in a shuttle box avoidance test (Tiwari et al. 2015), ETX (100, 200, or 250 mg/kg; i.p.) failed to improve T-maze spatial learning in Sprague–Dawley rats (Ponnusamy and Pradhan 2006), or to influence MWM performance in either 16-month-old APP/PS1, 3xTg-AD, or wild-type mice (30 mg/ml in drinking water) (Nygaard et al. 2015). Similarly, MWM performance failed to differ between 6-month-old WAG/Rij rats or Wistar rats chronically treated with ETX (300 mg/kg/day in drinking water for 17 days) and untreated controls. While ETX blunted the spatial memory impairment seen at 12 months in WAG/Rij rats, there was no effect in Wistar rats at the same age. Whether these results reflect a strain-specific mechanistic link between age-related memory decline and SWDs in WAG/Rij rats (Leo et al. 2019) remains to be established. One limitation of the current study is that the behavioral impact of ETX remains to be systematically characterized in the LE model of normal cognitive aging, but future studies in this model may provide important insights that further clarify the implications of the present findings. If ETX is effective at reducing SWD activity (as shown here) and is also shown not to improve cognition, such data would support the idea that SWD and cognitive performance are mechanistically dissociable.

Further work is similarly warranted when it comes to translational value. However, preclinical findings with AEDs like ETX highlight the complexity of this translational challenge. While ETX consistently suppresses SWDs in rodent models, its inconsistent effects on memory performance—particularly in APP/PS1 and 3xTg-AD mice—raise questions about whether SWD suppression alone is sufficient to confer cognitive benefit. This underscores the need for clinical trials not only to confirm whether SWD-targeting drugs reduce nonconvulsive spiking in MCI/AD patients, but also to determine whether such reductions translate into meaningful cognitive outcomes.

Another limitation of the current study is that the low power increases the risk of Type II error and limits the confidence with which null or modest findings can be interpreted. Like many features of aging in this and other species, the magnitude of effects, such as that of SWD activity, varies markedly between individuals, with nearly half of older subjects displaying no greater SWD frequency than much younger adult animals. Nevertheless, the data presented here clearly demonstrate an age-related increase in SWD activity in Long-Evans rats, consistent with prior reports.

Although SWD activity and spatial memory both differ in aged rats compared to young, the EEG and pharmacological results reported here indicate that these two prominent signatures of aging are unrelated to each other and mechanistically distinct. SWD activity is therefore likely independent of the well-documented age-related excitatory/inhibitory imbalance associated with poor cognitive outcome in aging. While the validity of spontaneous SWDs as a translational model of absence seizures remains debated (Wiest and Nicolelis 2003; Taylor et al. 2017, 2019), our findings suggest other avenues are likely to prove more fruitful in research on the neurobiology of cognitive aging and resilience.
